# Correlation of metastasis characteristics with prognosis in gastric mixed adenoneuroendocrine carcinoma: Two case reports

**DOI:** 10.1097/MD.0000000000009189

**Published:** 2017-12-15

**Authors:** Qiang Tang, Zili Zhou, Jinhuang Chen, Maojun Di, Jintong Ji, Wenzheng Yuan, Zhengyi Liu, Liang Wu, Xudan Zhang, Kang Li, Xiaogang Shu

**Affiliations:** Department of Gastrointestinal Surgery, Union Hospital, Tongji Medical College, Huazhong University of Science and Technology, Wuhan, China.

**Keywords:** gastric mixed adenoneuroendocrine carcinoma, metastasis, pathogenesis, prognosis, treatment

## Abstract

**Rationale:**

This article is aimed to retrospect the clinicopathological data of 2 cases of gastric MANENCs. MANEC is a rare biphasic tumor type that is coexistence of dual neuroendocrine and adenocarcinoma differentiation with each composing exceeding 30% volume. Gastric MANEC have just been reported anecdotally in the literature due to their rarity and heterogeneity. According to our study, these neoplasms have 3 different metastasis patterns: only adenocarcinomatous or neuroendocrine carcinoma and both of the 2 components. We first focus on the correlation of metastasis characteristics with prognosis in gastric MANEC, which may be potential implications for the choice of chemotherapy.

**Patient concerns:**

The 2 cases of patient shared several symptoms: epigastric discomfort, weight loss, hematemesis, or melena.

**Diagnosis:**

The 2 patients were diagnosis as MANEC based on the identification of histopathological analysis. In case 1, the poor differentiated adenocarcinoma accounted for 30%, the neuroendocrine part account for 70% and both of the 2 components metastasized to the lymph nodes, whereas in case 2, poorly differentiated adenocarcinoma accounted for 70%, the neuroendocrine part for 30% and only the glandular component invaded regional lymph nodes.

**Interventions:**

The first patient underwent laparoscopic radical gastrectomy and underwent adjuvant chemotherapy, combination of cisplatin, and etoposide successfully. The second patient received radical gastronomy, and did not receive any chemotherapy due to general weakness.

**Outcomes:**

The first patient is alive with no evidence of recurrence, and the second patient died 6 months after the operation.

**Lessons:**

The assessment of metastatic sites should be a routine pathological practice, which is crucial for clinical decision-making and the selection of management.

## 1 Introduction

The first description of the gastrointestinal tumor, which consists of dual neuroendocrine and exocrine differentiation, was reported by Cordier in 1924.^[1]^ After that, these compound tumor has been given many different pathologic defined names including argentaffin cell carcinoma, mcin-producing carcinoid, composite carcinoid, small or large cell undifferentiated carcinoma, and so on.^[2]^ In fact, these terms cannot epitomize the biological heterogeneity and natural behavior of mixed neoplasm adequately, which bring considerable confusion to pathologists and surgeons. In 2000, this mixed tumor was defined as mixed exocrine-neuroendocrine carcinomas by the World Health Organization (WHO) classification of endocrine tumors.^[3]^ And 10 years later, it was renamed as mixed adenoneuroendocrine carcinomas (MANECs) by 2010-WHO classification of neoplasm of the gastrointestinal tract. According to the classification, MANECs are absolutely defined as neoplasm morphologically with both gland-forming and neuroendocrine components, and each one represents at least 30% of the tumor.^[4]^

MANENCs, a biphasic tumor types, have just been reported anecdotally in the literature due to their rarity and heterogeneity. This neoplasm usually has a poor prognosis since both components are malignant. The pathogenesis and clinical features were reported with different characteristics controversially compared with adenocarcinoma or neuroendocrine carcinomas. Whether the biological behavior and characteristics of MANECs is more similar to neuroendocrine carcinoma or adenocarcinoma counterparts is still unknown.

Owing to the improved diagnostic techniques, tumors with neuroendocrine carcinoma occurring in the epithelial neoplasm have been increasingly reported in the stomach,^[5]^ biliary tract,^[6]^ colon-rectum,^[7]^ larynx, pancreas,^[8]^ and appendix with an increased incidence than previously believed. However, there are no previously reported cases of mixed adenocarcinoma endocrinologist carcinoma (MANEC) focusing on the correlation of the pattern of metastasis with prognosis in the English literature. The purpose of this article is to describe 2 patients with gastric MANEC that have different metastatic patterns, and finally received radical operation and adjuvant chemotherapy. From this, we can help to further understand of the characteristics of the disease raising the awareness of its diagnosis and contributing to optimize individualized therapy.

## 2 Methods

We searched the pathology records between 2015 and 2016, and found 2 cases meet the current diagnostic criteria of gastric MANEC as defined by the 2010 WHO classification in Wuhan Union Hospital. The histologicimmunohistochemical and clinical features were reassessed. The stage was defined according to the American Joint Committee on Cancer (AJCC) Cancer Staging Manual for carcinoma of the stomach, seventh edition. Human Subjects Protection Committee of the Huazhong University of Science and Technology had approved our study, and all participants gave informed consent.

### 2.1 Case presentation

#### 2.1.1 Case 1

A 64-years-old man who has a history of diabetes and mild hypertension was admitted to our hospital with epigastric discomfort after meals and with weight loss of 4 kg (originally 56 kg) within the last 2 months. Laboratory tests revealed increased levels of tumor markers (carcinoembryonicantige [CEA] 18.5 ug/L (<5 ug/L), cancerantigen 125 (CA125) 307.9 U/mL (<35 U/mL) and with milder anemia]. The patient underwent esophagogastroscopy, which revealed a ulcer lesion sizing 4.0 to 5.0 cm at the cardia. Neoplasm multisite biopsy specimen revealed poorly differentiated adenocarcinoma with scattered signet ring cells. The abdominal contrast-enhanced computed tomography (CT) showed that the posterior wall of the gastric was stiffness, thickening, with adjacent lymph node enlargement, and no evidence of distant metastases (cT3N1M0 stage IIIA). The patient underwent laparoscopic radical gastrectomy with Roux-en-Y esophagojejunostomy, which lasted about 4 hours. We found a 4 to 5-cm solid plasma that invaded to the serous of the gastric wall and multiple enlarged lymph nodes in the adjacent region.

**Figure 1:**
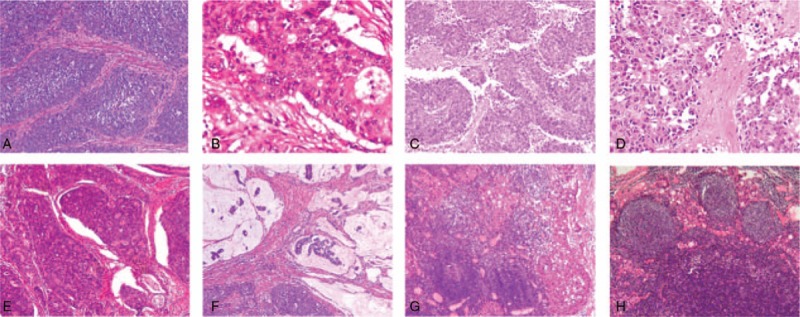
In case 1: histology of the primary mixed adenoneuroendocrine carcinoma (MANEC) in stomach (A, B). The lymph nodes infiltrated by both of 2 components (C, D). In case 2: On hematoxylin–eosin staining of primary tumor (E, F). In the metastatic lymph nodes, the adenocarcinoma was predominant (G, H).

Histopathological analysis reported an ulcerative mass, which was diagnosed as MANEC. The differentiated adenocarcinoma accounted for 30%, whereas the typical neuroendocrine small neuroendocrine cancer cells irregularly distributing in the nest accounted for 70%, which had scarce cytoplasm and coarse chromosome. The tumor involved subserosal tissues and perineural invasion, with metastases in 10 of the 28 regional lymph nodes. Vascular lumina, spleen, and omentum were not found being infiltrated (pT4aN1M0). Immunohistochemistry showed that the adenocarcinoma components were middle to strongly positive for protein kinase C (PKC), but negative for synaptophysin (Syn), CD56, and chromogranin A (CgA). The Ki67 labeling index was 70%. While the neuroendocrine cancer cells were positive for CgA, Syn and weakly for CD56, CDX2. The lymph nodes at lesser curvature gastric body were infiltrated by both of 2 components (Fig. 1A–D). After the operation, the patient had no complications occurred and uneventfully recovered. He underwent adjuvant chemotherapy successfully, combination of cisplatin and etoposide at the 6-month follow-up. He was alive with no evidence of recurrence.

#### 2.1.2 Case 2

A 52-years-old man was referred to our hospital with several symptoms: weight loss, hematemesis and melena. No obvious abnormality was found in the physical examination and laboratory tests. The esophagogastroscopy revealed that a mess 4.5 to 5.0 cm at the greater curvature of the antrum. Multisite biopsy specimen revealed poorly differentiated carcinoma with appearance of scattered signet ring cells. The abdominal contrast-enhanced CT showed that the stomach wall was stiffness, and a mess 5.0 to 6.0 cm at greater curvature of the antrum, with adjacent lymph node enlargement (cT3N1M0 stage IIIA).

The patient underwent radicalgastronomy. We found a 5-cm diffuse mass, and multiple enlarged lymph nodes in the adjacent region. Histopathologic analysis reported that the poorly differentiated adenocarcinoma accounted for 70%, and the most of cells showed signs of signet-ring cell carcinoma. Neuroendocrine cancer cells are irregularly distributing in the nest (not >30%). The tumor invaded muscle layer and subserosal tissues. Perineural, vascular lumina were found being infiltrated. Five of the 13 regional lymph nodes showed metastasis and no distant metastasis detected (pT4aN1M0).

Immunohistochemistry showed that PCK (+), CD56 (−), Syn (−), CgA (−), Ki-67 40% for adenocarcinoma cells and PCK (+), CD56 (+), CgA (+), Syn (−/+), Ki-67 25% for neuroendocrine carcinoma cells. In the metastatic lymph nodes, the glandular component was predominant and the neuroendocrine carcinoma cells were not seen (Fig. 1E–H). After the surgery, the patients did not receive any chemotherapy due to general weakness and died 6 months after the operation.

## 3 Discussion

Gastroenteropancreatic neuroendocrine tumors (GEP-NETs), derived from neuroendocrine cells, were first proposed by pathologist Siegfried Oberndorfer in 1907.^[9]^ According to a multicenter analysis of the epidemiology surveillance, annual incidence of G-NETs is ranging from 0.3 to 5.25 cases per 100,000 people in the world.^[2]^ The WHO-2010 classification scheme updated its classification system and classified all ENETS into the following histological categories: neuroendocrine tumor grade 1 (NET G1), NET G2, and NET G3 (NEC), mixed endocrinologist carcinoma (MANEC).^[10]^ In cytology, the classification scheme depended on the differentiation (number of nuclear division and Ki-67 proliferation index), which means the extent of malignant cells being resemble to normal cells. Tumor morphology and Ki-67, an indicator proliferation rate was defined as the critical prognostic factors.

According to the grade of differentiation and malignancy of the 2 components, MANECs was further divided into following subtypes: high-grade malignant MANEC, intermediate-grade malignant MANEC, and low-grade malignant mixed endocrinologist tumor. This simpler proliferation-based ENETS/WHO 2010 classification system provides prognostic information, but not yet validated to predict relapse after surgical resection. Thus, site-specific histologic features combined with proliferative grading may more adequately epitomize the biological heterogeneity of gastric mixed neoplasm requiring more aggressive therapy.^[11,12]^

Despite the constant new cases reported, the histological origin of gastric MANEC is remains unclear yet. An increasing number of pathologist proposed 2 main theories to explain the emphasis morphology of this neoplasm.^[13,14]^ On the one hand, the 2 components of MANECs descended from 2 different cell lines. The adenocarcinoma cells were originated in pluripotent stem cells, and neuroendocrine cancer cells were embryonic neural cells. On the other hand, MANEC is originated from single endoderm pluripotent stem cells, which are affected by hormones, local microenvironment and instable genome, during the process of tumor genesis and development, which eventually lead to 2-way or multidirectional differentiation.^[7]^ In most studies, adenocarcinoma and neuroendocrine components of the MANEC are cross-mixed together, only few cases of the 2 components are closely linked without mixing (as so-called collision tumor), suggesting that most cases may originate from multipotential stem cells that went through multidirectional differentiation in the process of tumor generation and development. Zhang et al^[15]^ reported that in addition to the components of carcinoma and neuroendocrine carcinoma, the mixed carcinoma also contained squamous cell carcinoma, which may be more supportive of the tumor originated in the multipotential stem.^[15]^ So we are more convinced that the occurrence mechanism of mixed carcinoma belongs to the later theory.

With the development of molecular biology, many scholars have focused on the molecular mechanism of MANEC pathogen. Loss of heterozygosity and mutational analysis of chromosomes indicates that both of the components share similar mutational profiles and multistep progressions from common precursor lesion. Several shared mutations (P53, KRAS) in the subsequent exome sequencing revealed a clonal relationship between the adenocarcinoma and neuroendocrine component. A further study revealed that methylation of Ras association domain family 1 isoform (RASSF1A) has an inverse correlation with mutations of the KRAS and BRAF genes in such neoplasm. Methylation (p16^INK4A^ and hMLH1) may be one of the molecular pathogenesis of the gastric MANEC.^[16]^ While the neuroendocrine component showed higher frequency of chromosomal abnormalities than the adenocarcinoma.^[15]^ Comparative exome sequencing neuroendocrine carcinoma and adenocarcinoma component found that mutations of SMARCA4 was only observed in neuroendocrine component, which suggested that SMARCA4 is probably responsible for the transformation neuroendocrine phenotype. Multiple driver genes (*ATM*, *CTNNB1*, *ERBB4*, *Janus Kinase 3*, *KDR*, *KRAS*, *RB1*) were frequently observed in the poor differentiated neuroendocrine component.^[14]^ Further studies are needed to reveal the potential molecular pathogen of MANEC, which may fundamental importance to bring in innovative and targeted stratified therapeutic option.

Metastasis is the important characteristics of malignant gastric cancer but also the most important risk factors leading to the death of patients. Gastric MANEC were always diagnosed with lymphnodes metastasis and liver metastasis.^[14,16]^ There was no consensus on the metastatic patterns in most of the experiments reported to date. Other basis of the histologic type, the pattern of metastases can be divided into: neuroendocrine carcinoma or adenocarcinoma component only, the adenocarcinoma and neuroendocrine carcinoma, squamous cell carcinoma or coexist with adenocarcinoma or neuroendocrine carcinoma (seen in few cases). A study revealed that lymphnodes and liver metastasis were usually invaded by the neuroendocrine carcinoma rather than adenocarcinoma component about 67%.^[15,17,18]^ Only 24% of the metastasis tumors were adenocarcinoma component^[19]^ (as seen in the Table 1). In our recent literature, the 2 cases belong to the latter pattern. In the first case, lymph nodes of metastasis contained mixed neoplasm type, which are both gland-forming and neuroendocrine neoplasm. While in the second case, the adenocarcinoma cell type mainly occupied the tumor and infiltrated the peripheral lymph nodes. And among the metastatic neuroendocrine tumor, the rate of poorly differentiated neuroendocrine carcinomas is up to 90%.^[20]^ Beom Su Kim reported that the degree of differentiation and perineural or vascular invasion may be an independent risk factors that influence lymph node or distance metastasis in MANEC.

**Table 1:**
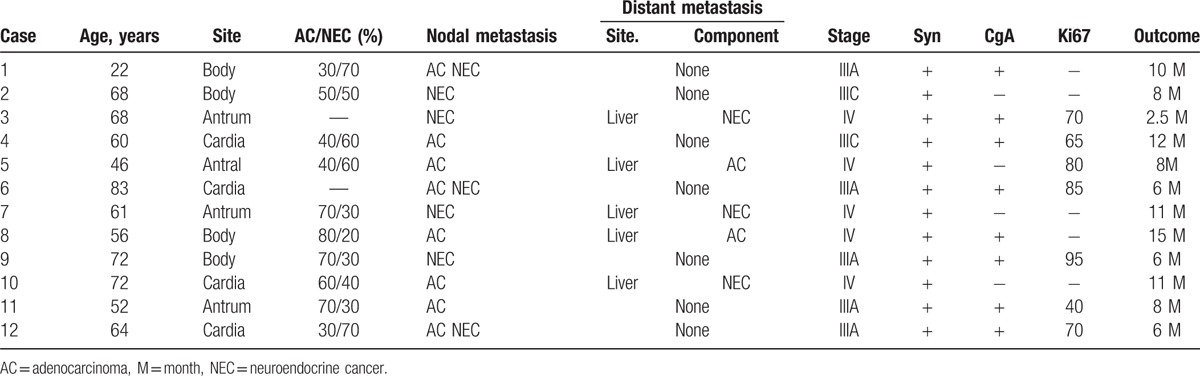
Clinicopathologic characteristics of the 12 cases with gastric MANEC in the literature.

Obviously, the different modes of metastasis have a vital role in the diagnosis and treatment (including primary and metastatic lesions) and assessing the prognosis. Traditionally, the volume percentage of the 2 components was believed to determine the clinical course of mixed carcinoma. While, a recent retrospective analysis reported that the outcome of MANEC depends on the predominant component,^[21]^ rather than the proportion of each component. Simply because that smaller percentage volume can still metastasize and have a significant impact on the patients’ outcome (seen in Table 1). Mostly authors sustain that the characteristics of neuroendocrine part have a considerable impact on the clinical behavior of the MANEC.^[22]^ And in most literature reported, the lymph node and liver metastatic were always neuroendocrine part rather than adenocarcinoma one. Because neuroendocrine carcinoma were usually poorly differentiated, and more aggressive compared with the adenocarcinoma. Maria Scardoni concluded that there are more multiple driver gene mutations such as *ATM*, *ERBB4*, *KDR/VEGFR2*, *JAK3*, and *TP53* gene^[14]^ in the neuroendocrine component. Recently, hypermethylated tumor specific RASSF1A is confirmed to have a closely relationship with lymph node metastases in well differentiated NETS.^[23]^ While others contained that adenocarcinoma component may influence the prognosis in the well-differentiated neuroendocrine tumor of MANEC. A study reported that the mortality rate of the histological types that combination of low-grade adenocarcinoma with unfavorable neuroendocrine tumors was obviously higher than that of combination of high grade glandular tumor types with low-grade neuroendocrine tumors.^[20]^ It showed that the glandular component would be the main driving force of the cancer progression, rather than the neuroendocrine part in this carcinoma type. These were still lack of a large number of clinical studies and epidemiological analysis.

The fact that whether the glandular or the neuroendocrine component is the critical factors of cancer progression is remains unclear, so as to the established standard treatment paradigm treatment for metastatic cancer. Although the most recently WHO classification suggests that the treatment of MANECs is similar to the common adenocarcinoma. Lee et al^[5]^ put forward that the therapy should focus on the more aggressive histology component of the tumor, because the clinical prognosis of this mixed carcinoma follows that of a more invasive component. In cases of low-grade malignant neuroendocrine component with an unfavorable adenocarcinoma, the therapy should primarily focus on the exocrine component. Contrarily, the treatment should focus on the neuroendocrine component.

Recently, the surgical treatment-based assisted with comprehensive treatment such as radiotherapy or chemotherapy was widely accepted by most clinicians.^[24–27]^ Palliative surgery or liver segment resection are believed to relieve clinical symptoms and prolong the survival, especially for the patients accompanied with distant metastasis. Current clinical trials reported that capecitabine combined with cisplatin can increased the 5-year overall survival rate and relief the symptoms in gastric neuroendocrine tumors and is recommended as the most favorable regime for the advanced cancer.^[27]^ Somatostatin analogue (octreotide) has been used in the treatment of advanced gastrointestinal neuroendocrine tumors, which can effectively control carcinoid syndrome and inhibit tumor development by regulating the signals of proliferation and apoptosis.^[27]^ In recent years, increasing understanding of tumor biological behavior and molecular basis of tumor has led to application of targeted therapy for this carcinoma. The neuroendocrine tumors guidelines has recommended targeted therapies as second-line medication such as sunitinib, everolimus, and bevacizumab that have a positive effect in the management of carcinoma.^[10]^

The prognosis of carcinoma involves many clinical and pathological factors, such as the tumor size, stage, histological differentiation, metastasis and reasonable treatment. Though the prognosis of gastric MANEC is not well-defined, the previous available data revealed that high-grade MANEC were usually lead a worse outcome than that of normal gastric adenocarcinoma.^[14]^ Unfortunately, about 67% of patients were diagnosed with stage III and IV.^[28]^ La Rosa et al. reported that 92% of MANECs had lymphatic invasion, vascular and perineural invasion, consistent with the other aggressive biological characteristics. Jiang reported that mixed tumors have a longer overall survival and median progression free survival than that of pure large cell NEC, which is considered as the most aggressive subgroup. However, a previous study about colorectal MANEC reported that there was no statistical significant differences in survival rate between colorectal MANECs and pure neuroendocrine tumors,^[29]^ suggested that tumor site may be a prognostic factors. The intermediate grade malignant MANEC, in which the adenocarcinoma component is biologically more aggressive than the neuroendocrine one, show an equivalent outcome than the adenocarcinoma. But the low-grade malignant MANEC have excellent prognosis, because no evidence of recurrence was reported in postoperative observation recently. A recently research showed a tendency of grim outcome with the increase of proportion of the neuroendocrine component and grade. It revealed that patients with lymph node or liver metastasis consist both neuroendocrine component and adenocarcinoma component have a shorter median survival time than that of the metastasis lesion with neuroendocrine component or adenocarcinoma component only, and metastasis lesions with adenocarcinoma component have a favorable prognosis than the other 2.^[30]^ So we speculated that the pattern of metastasis may be a evaluating indicator for the prognosis.

In clinical pathological practice, lymph node distant metastases are not routinely assessed by histology, when the primary tumor is known. The cases reported in this present paper exhibited 2 different patterns of dissemination, which may be potential implications for the choice of chemotherapy and evaluation of prognosis. The further evaluation of metastatic sites should be a routine pathological practice that is crucial for clinical decision-making and the selection of managements.

## 4 Conclusions

The current article is aimed to retrospect the clinicopathological data of 2 cases of gastric MANENCs. MANEC is a rare biphasic tumor type that is coexistence of dual neuroendocrine and adenocarcinoma differentiation with each composing exceeding 30% volume. Gastric MANECs have just been reported anecdotally in the literature due to their rarity and heterogeneity. According to our study and the literature, these neoplasms have different metastasis characteristics, which may be potential implications for the choice of chemotherapy. The assessment of metastatic sites should be a routine pathological practice, which is crucial for clinical decision-making and the selection of managements.
